# Focal Hepatic Hot Spot Sign Mimicking Active Extravasation on CT Angiography in Superior Vena Cava Syndrome: A Case Report

**DOI:** 10.5811/cpcem.64768

**Published:** 2026-10-13

**Authors:** R. Gentry Wilkerson, Vanesa Brecher

**Affiliations:** * University of Maryland School of Medicine, Department of Emergency Medicine, Baltimore, Maryland; † Philadelphia College of Osteopathic Medicine, Philadelphia, Pennsylvania

**Keywords:** focal hepatic hot spot sign, hot quadrate sign, superior vena cava syndrome, hepatic pseudolesion, case report

## Abstract

**Introduction:**

Caused by portosystemic venous shunting, the focal hepatic hot spot sign (or hot quadrate sign) is a radiologic finding most commonly associated with superior vena cava (SVC) obstruction. On contrast-enhanced imaging, this finding may mimic active hepatic extravasation or focal hepatic pathology, posing a diagnostic challenge in the emergency department (ED).

**Case Report:**

We report a case of a 70-year-old man with progressive cognitive decline and hypoxemic respiratory failure requiring intubation. Computed tomography (CT) angiography of the chest demonstrated a focal area of intense contrast enhancement in the left hepatic lobe, initially concerning for active extravasation. Further review of the imaging revealed a right upper lobe lung mass and a large mediastinal mass causing high-grade SVC stenosis. The hepatic finding was subsequently identified by the reading radiologist as a focal hepatic hot spot sign representing a vascular pseudolesion due to collateral venous flow. The patient was diagnosed with SVC syndrome secondary to malignancy and underwent chemotherapy before transitioning to comfort care.

**Conclusion:**

The focal hepatic hot spot sign represents a hepatic pseudolesion resulting from altered venous return and collateral circulation, most commonly in the setting of SVC obstruction. In the ED, this finding may be misinterpreted as hemorrhage or focal hepatic pathology, potentially leading to unnecessary diagnostic or therapeutic interventions. Recognition of the focal hepatic hot spot sign is essential for emergency physicians to avoid misdiagnosis and identify underlying central venous obstruction. Awareness of this imaging pitfall can improve diagnostic accuracy and guide appropriate management in patients undergoing contrast-enhanced CT imaging.

## INTRODUCTION

The focal hepatic hot spot sign (or hot quadrate sign) is a radiologic finding characterized by focal enhancement in segment IV of the liver due to portosystemic venous shunting.^1^ Although most commonly associated with superior vena cava (SVC) obstruction, it may be misinterpreted on contrast-enhanced imaging as active hepatic extravasation or focal hepatic pathology.^2^ Given the widespread use of computed tomography (CT) angiography in the emergency department (ED) for evaluation of undifferentiated patients, emergency physicians are increasingly likely to encounter incidental hepatic findings, making familiarity with this imaging finding essential to avoid diagnostic error. We present a case in which this finding initially raised concern for intra-abdominal hemorrhage but ultimately led to the diagnosis of SVC syndrome from an underlying malignancy.

## CASE REPORT

A 70-year-old man with a past medical history of hypertension, coronary artery disease with previous coronary artery stent placement, ischemic cardiomyopathy with a previously documented ejection fraction of 25% to 30%, chronic obstructive pulmonary disease, and continued heavy cigarette smoking was brought in by family members due to worsening cognitive decline and frequent falls over the previous month. The family reported a fall 3 weeks prior to ED presentation with persistent difficulty with moving his left arm since the fall.

At triage, his vital signs were as follows: blood pressure, 127/84 mm Hg; heart rate, 82 beats per minute; respiratory rate, 16 breaths per minute; temperature, 36.3 °C; and oxygen saturation level, 97% on room air. He was somnolent with snoring respirations. The rest of his cardiopulmonary exam was unremarkable. There was 2+ pitting edema of the bilateral lower extremities. He would waken briefly to strong tactile stimuli and was able to follow brief, simple commands, but he was not able to speak. There was no obvious focal neurologic deficit. During the patient’s ED visit, he became increasingly hypoxic requiring oxygen supplementation by nasal cannula and then high-flow nasal cannula, and eventually intubation with mechanical ventilation.

A CT angiogram of the chest demonstrated a focal area of intense contrast enhancement within the medial aspect of the left hepatic lobe (Image 1), initially raising concern by the clinical team for active contrast extravasation. Additional review of the imaging revealed a 3.6 × 2.8 cm right upper lobe lung mass in the setting of fibrocystic and emphysematous lung changes, as well as a large 6.8 × 6.9 × 6.9 cm mediastinal mass causing significant mass effect on the right main pulmonary artery and SVC (Image 2), resulting in high-grade stenosis and findings consistent with SVC syndrome. The focal hepatic enhancement was subsequently identified by the radiologist as a vascular pseudolesion representing collateral venous flow to segment IV of the liver, consistent with the focal hepatic hot spot sign. The patient underwent a 3-day induction of chemotherapy with atezolizumab/carboplatin/etoposide. Due to persistence of acute hypoxemic respiratory failure, the patient’s family elected to change the patient’s code status to “do not resuscitate” and made him comfort care measures only. The patient was transferred to an inpatient hospice unit on hospital day 14.

*CPC-EM Capsule*

What do we already know about this clinical entity?

*The focal hepatic hot spot sign is a vascular pseudolesion due to portosystemic shunting, most commonly associated with superior vena cava (SVC) obstruction.*

What makes this presentation of disease reportable?

*The focal hepatic hot spot sign mimicked active extravasation on computed tomography angiography and contributed to recognition of malignant SVC obstruction.*

What is the major learning point?

*Intense enhancement of hepatic segment IV can reflect collateral venous flow from SVC obstruction rather than hepatic hemorrhage or a true liver lesion.*

How might this improve emergency medicine practice?

*Recognizing this sign can prevent unnecessary intervention and prompt evaluation for potentially life-threatening central venous obstruction.*

## DISCUSSION

The focal hepatic hot spot sign (or hot quadrate sign) is a radiologic finding characterized by focal hyperenhancement of segment IV of the liver (the quadrate lobe) during arterial and early portal venous phases of contrast-enhanced imaging.^1^ This finding represents a hepatic pseudolesion caused by altered venous return and portosystemic shunting, most commonly in the setting of SVC obstruction.^3^ This finding has also been described on technetium-99m sulfur colloid liver scans, further supporting its underlying mechanism of altered hepatic perfusion due to portosystemic shunting.^2^

In cases of SVC obstruction, contrast injected into the upper extremity is diverted through collateral venous pathways, including the internal thoracic and paraumbilical veins, ultimately reaching the liver via the veins of Sappey and preferentially perfusing the left hepatic lobe.^3^ The resulting localized increase in contrast delivery produces the characteristic focal enhancement seen on CT. Recognition of this phenomenon is essential because it reflects underlying central venous pathology rather than intrinsic hepatic disease. Hepatic pseudolesions related to altered venous drainage may be present in up to 20% of contrast-enhanced CT studies, although the focal hepatic hot spot sign represents a specific and clinically meaningful subtype.^4^

In the ED, the focal hepatic hot spot sign is particularly important because it may be misinterpreted as active contrast extravasation or focal hepatic pathology on contrast-enhanced CT, especially when identified incidentally during evaluation for other acute conditions.^2^ Such misinterpretation may prompt unnecessary diagnostic workup, invasive procedures, or inappropriate clinical management. Recognition of this pseudolesion should instead prompt careful evaluation for central venous obstruction, particularly SVC syndrome, which may present with subtle or nonspecific findings early in its course. Classic clinical findings of SVC syndrome include facial and upper extremity swelling, dyspnea, and the presence of the Pemberton sign, in which elevation of the arms results in facial plethora and venous distention; however, these findings may be absent early in the disease process.^5^ In this case, the initial concern for hepatic extravasation was redirected after identification of mediastinal pathology causing significant SVC compression. Recognition of this imaging sign allows emergency physicians to appropriately redirect diagnostic evaluation, avoid unnecessary interventions, and expedite identification of the underlying etiology.

The differential diagnosis for focal hepatic hyperenhancement on contrast-enhanced CT includes both true hepatic lesions and pseudolesions. Entities such as hemangioma, focal nodular hyperplasia, and hepatocellular carcinoma may demonstrate hyperattenuation and can mimic this appearance, particularly when imaging is obtained during arterial phase acquisition.^6^ However, unlike true lesions, the focal hepatic hot spot sign does not produce mass effect and is typically localized to segment IV near the falciform ligament. Correlation with clinical findings and careful evaluation for associated features of central venous obstruction, such as mediastinal masses, venous collateralization, or SVC narrowing, can aid in distinguishing this pseudolesion from true pathology. Recognition of these distinguishing features is critical in the emergency setting, where rapid interpretation of imaging findings directly impacts diagnostic accuracy and subsequent management decisions.

## CONCLUSION

The focal hepatic hot spot sign is an important imaging finding that may be encountered incidentally on contrast-enhanced CT in the ED. Recognition of this pseudolesion is critical to avoid misdiagnosis as hepatic hemorrhage or focal liver pathology. In the appropriate clinical context, this finding should prompt evaluation for central venous obstruction, including SVC syndrome. For emergency physicians, recognition of this sign can prevent misdiagnosis, avoid unnecessary intervention, and serve as an early imaging clue to life-threatening central venous obstruction.

## Figures and Tables

**Image 1:**
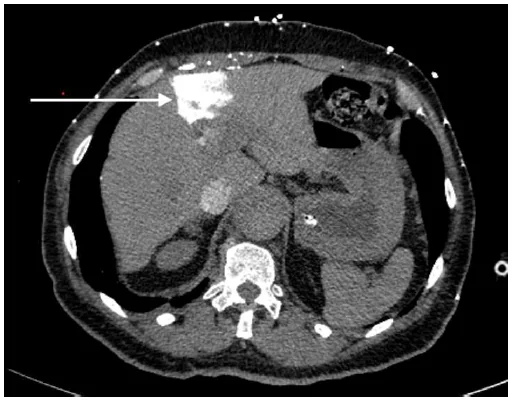
Axial contrast-enhanced computed tomography angiography demonstrates a focal area of intense hyperenhancement within segment IV of the liver (white arrow), initially concerning for active contrast extravasation.

**Image 2:**
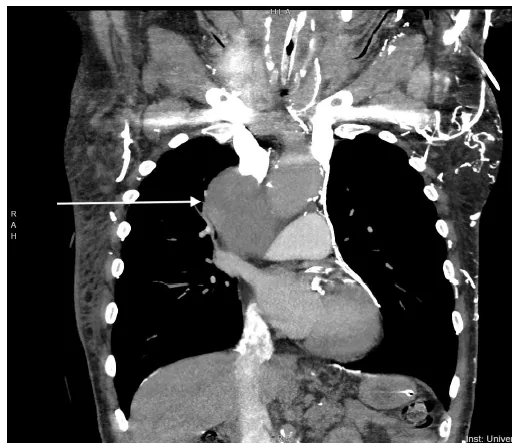
Coronal contrast-enhanced computed tomography demonstrates a large mediastinal mass (arrow) causing significant compression of the superior vena cava (SVC), resulting in SVC syndrome and the development of collateral venous pathways responsible for the focal hepatic hot spot sign.
